# *In vitro* protocol demonstrating five functional steps of trained immunity in mice: Implications on biomarker discovery and translational research

**DOI:** 10.1016/j.celrep.2025.116202

**Published:** 2025-09-26

**Authors:** Maria González-Pérez, Jana Baranda, Leticia Pérez-Rodríguez, Patricia Conde, Carlos de la Calle-Fabregat, Marcos J. Berges-Buxeda, Alexander Dimitrov, Javier Arranz-Herrero, Sergio Rius-Rocabert, Alessia Zotta, Ana Dopazo, Nikita Poddar, Xuedi Wang, Estanislao Nistal-Villán, Raphaël Duivenvoorden, Joren C. Madsen, David L. Williams, Dan Hasson, Daniel Lozano-Ojalvo, Florent Ginhoux, Luke A.J. O’Neill, Jordi Ochando

**Affiliations:** 1 National Microbiology Center, National Institutes of Health Carlos III, Madrid, Spain; 2 Department of Pharmaceutical Sciences, CEU-San Pablo University, Madrid, Spain; 3 Department of Oncological Sciences and Tisch Cancer Institute, Icahn School of Medicine at Mount Sinai, New York, NY, USA; 4 Institut National de la Santé Et de la Recherche Médicale (INSERM) U1015, Gustave Roussy Cancer Campus, Villejuif, France; 5 School of Biochemistry and Immunology, Trinity Biomedical Sciences Institute, Trinity College Dublin, Dublin, Ireland; 6 Genomics Unit, National Cardiovascular Center (CNIC), Madrid, Spain; 7 Tisch Cancer Institute Bioinformatics for Next Generation Sequencing, Icahn School of Medicine at Mount Sinai, New York, NY, USA; 8 Department of Nephrology, Radboud University Medical Center, Nijmegen, the Netherlands; 9 Biomolecular Engineering and Imaging Institute, Icahn School of Medicine at Mount Sinai, New York, NY, USA; 10 Center for Transplantation Sciences and Division of Cardiac Surgery, Department of Surgery, Massachusetts General Hospital, Boston, MA, USA; 11 Department of Surgery and Center for Inflammation, Infectious Disease and Immunity, East Tennessee State University, Johnson City, TN, USA; 12 Immunology Institute, Icahn School of Medicine at Mount Sinai, New York, NY, USA; 13 These authors contributed equally; 14 Lead contact

## Abstract

We developed an *in vitro* methodology to study trained immunity using murine bone-marrow-derived macrophages stimulated with β-glucan and lipopolysaccharide (LPS). Longitudinal analysis of interleukin (IL)-6 and tumor necrosis factor (TNF) production demonstrates that trained macrophages secrete higher cytokine levels following primary stimulation with β-glucan compared to unstimulated macrophages (step 1). After a resting period, trained macrophages return to basal levels of cytokine production (step 2) but rapidly produce enhanced levels of IL-6 and TNF after secondary stimulation with LPS, compared to macrophages individually stimulated with either β-glucan (step 3) or LPS (step 4) alone. The combined cytokine production of macrophages after single stimulation with β-glucan (stimulus 1) and LPS (stimulus 2) is significantly lower than the cytokine levels produced by trained macrophages sequentially stimulated with both β-glucan and LPS (stimulus 1 + 2) (step 5). These results experimentally reproduce the distinctive functional stages that macrophages undergo during the training process.

## In brief

González-Pérez et al. developed an *in vitro* method to induce trained immunity using murine bone-marrow-derived macrophages. Standardization of training protocols using experimental mouse models is critical for advancing our understanding of the mechanisms that regulate innate immune-memory responses and develop therapies that target trained macrophages under pathological conditions.

## Graphical Abstract

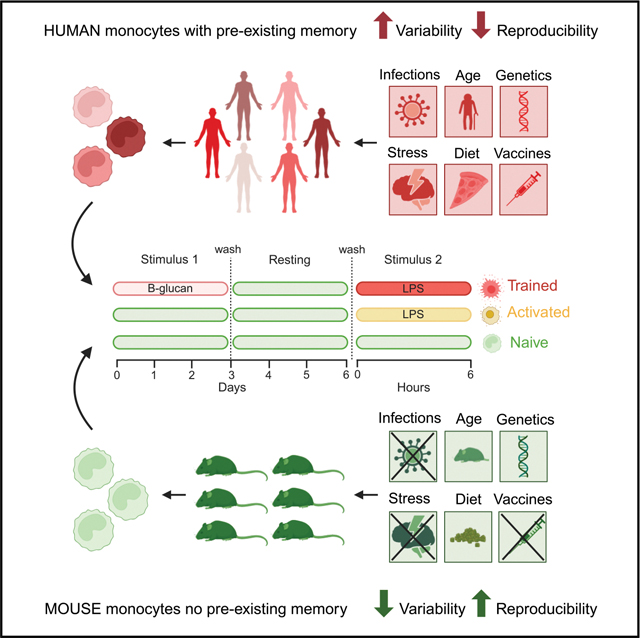

## INTRODUCTION

Innate immune cells orchestrate the front line against infectious agents by sensing pathogen-associated molecular patterns (PAMPs) through pattern-recognition receptors (PRRs).^1^ Stimulation of PRRs on myeloid cells induces a rapid inflammatory response that helps contain the infection and initiates adaptive-immunity-protecting mechanisms via antigen presentation (signal 1), co-stimulation (signal 2), and cytokine secretion (signal 3).^2^ Activated T cells then acquire the capacity to proliferate and undergo clonal expansion to increase the number of antigen-specific effector cells against the invading pathogen. Once the pathogen is eliminated and the inflammatory response is resolved, a subset of memory T cells persists, triggering enhanced immune responses upon re-exposure to the same infectious agent. While antigen-specific memory is an exclusive function of adaptive immune cells undergoing gene rearrangement and Variable Diversity Joining rearrangement recombination, innate immune cells are able to acquire “memory-like” functional properties. This adaptive program of the innate immune system is induced after a primary exposure to a pathogenic stimulus and confers a protective immune response against a secondary challenge in a T cell-independent manner. Systemic resistance against reinfection is an acquired functional program of the innate immune system conserved across species, although the mechanisms underlying memory responses in innate immune cells were largely unknown until recently.

In 2011, Mihai Netea and collaborators introduced the concept of “trained immunity” to describe the ability of the innate immune system to “remember” previous pathogenic encounters and enhance protection against secondary infections through increased production of pro-inflammatory cytokines. To experimentally validate this concept, Netea and colleagues administered non-lethal doses of *Candida albicans* to different congenic mouse strains. Once fungal loads and circulating cytokine concentrations were no longer detectable (resting period), mice were administered a second lethal dose of *C. albicans* and survival rates were assessed. Survival analysis demonstrated that trained mice were protected against reinfection through macrophage-dependent mechanisms and increased serum levels of interleukin (IL)-6 and tumor necrosis factor (TNF). These findings aligned with previous reports showing that mice inoculated with low-virulence *C. albicans* were protected not only from fungal reinfection but also against unrelated bacterial infections via macrophage activation and cytokine production.^3–5^ The results also emphasized the value of experimental mouse *in vivo* studies with translational relevance for human immunology and the integration of congenic mouse strains with targeted gene mutations, such as RAG-1-deficient or nude mice,^6,7^ to dissect unknown mechanisms of the innate immune-memory responses.

To further investigate the mechanisms that regulate protective innate immune-memory responses, trained-immunity-inducing *in vitro* protocols needed to be developed as proof of concept.^8,9^ Simplified *in vitro* methods without the complexity of a multicellular environment where multiple signaling pathways are activated simultaneously offer significant advantages for studying unknown regulatory mechanisms of trained immunity. These include the precise sequential stimulation of innate immune cells at different time intervals to evaluate functional adaptations and identify key molecules that participate in the induction and maintenance of trained immunity. Although less physiologically relevant, *in vitro* methods are critical for uncovering the underlying mechanisms that regulate trained immunity, providing insights that are difficult to obtain from *in vivo* models. This is particularly relevant in trained-immunity studies, where specific cell markers are urgently needed to accurately identify and isolate monocyte-derived cells that receive sequential training stimuli *in vivo*, within heterogeneous subsets of non-trained cells. To reproduce the *in vivo* model of protective immunity and recapitulate macrophage-dependent mechanisms that induce increased serum levels of inflammatory cytokines after *C. albicans* reinfection, human monocytes isolated from peripheral blood mononuclear cells (PBMCs) were stimulated with β-glucan purified from *C. albicans in vitro*. β-glucan is recognized by the C-type lectin receptor dectin-1 expressed on the cell surface of monocytes,^10,11^ and, following a resting period, β-glucan-stimulated monocytes secreted enhanced levels of IL-6 and TNF upon secondary stimulation with bacterial lipopolysaccharide (LPS) compared to non-β-glucan treated macrophages. Mechanistically, increased cytokine secretion was associated with transcriptional upregulation of *Il6* and *Tnf* mRNA, which correlated with epigenetic reprograming of H3K4me3 and was partially inhibited by the histone methyltransferase inhibitor MTA.^12^ Further studies revealed that β-glucan stimulation promotes aerobic glycolysis and cholesterol synthesis necessary to induce the production of metabolites that regulate histone modifications at pro-inflammatory gene loci.^13,14^ These modifications increase chromatin accessibility and enhance gene transcription upon secondary stimulation, demonstrating that trained immunity is a functional program driven by metabolic, transcriptomic, and epigenetic reprogramming of macrophages.^15^

Development of *in vitro* models to study trained immunity with human PBMCs has been critical in determining the training capabilities of additional PAMPs, such as the live attenuated form of the bacterium *Mycobacterium bovis*, present in the bacillus Calmette-Guérin (BCG) vaccine. In addition, this *in vitro* approach revealed that trained immunity is also induced by endogenous damage-associated molecular patterns (DAMPs), such as Oxidized Low-Density Lipoprotein and uric acid crystals, through metabolic and epigenetic reprogramming.^16,17^ Interestingly, while the mammalian target of rapamycin (mTOR) integrates multiple endogenous and exogenous signals that determine the outcome of the innate immune response,^18,19^ macrophage stimulation with β-glucan, BCG, and OxLDL induces trained immunity through mTOR-dependent mechanisms.^20^ Altogether, the development of *in vitro* models using human PBMC-derived monocyte protocols has been pivotal in advancing the field of trained immunity, indicating that conserved epigenetic reprogramming mechanisms occur downstream of mTOR in trained macrophages independently of the inciting stimuli.

Significant progress in the fundamental understanding of trained immunity led to a consensus update on the definition of training in 2021 to establish standardized criteria that distinguish trained immunity from other functional innate immune programs.^21^ The revisited definition of training included the previously described capability of trained cells to mount faster and greater responses against secondary challenges (step 4). However, the main difference between innate immune cells undergoing different adaptive programs focused on functional status prior to secondary challenge. Unlike priming, where immune activation persists, trained innate immune cells must return to a basal functional state after removal of the primary stimulus. This revised definition of trained immunity emphasized the importance of measuring the functional state of innate immune cells during the primary stimulation (step 1), as well as after the removal of the initial stimulus or resting period (step 2), to demonstrate that the functional immune response following secondary stimulation is more potent than the primary stimulation (step 3). Therefore, the impact of a second challenge in primed cells is often additive, while, in trained innate immune cells, it is synergistic with the original stimulus (step 5). The models used to study trained immunity must reflect the definition of this memory program of innate immune cells, and experimental methods should reproduce the different functional stages that macrophages undergo during the training process.

The mechanisms involved in innate memory responses depend on epigenetic remodeling. While mechanisms for epigenetic remodeling during trained immunity have recently been reviewed,^22^ the duration and maintenance of chromatin-driven innate memory responses remain largely unknown. In the context of infectious diseases, BCG vaccination is associated with increased production of inflammatory cytokines after LPS stimulation in blood mononuclear cells up to 1 year after vaccination.^23^ Long-term epigenetic reprogramming of myeloid cells and their precursors in the bone marrow is responsible for the duration of trained immunity, which is also induced by β-glucan administration.^24,25^ Notably, SARS-CoV-2 infection and vaccination against COVID-19 and flu have been reported to induce trained immunity in innate immune cells and their progenitors,^26–28^ affecting the innate immune-memory state of millions of individuals worldwide. In addition to previous infections and vaccination history, epigenetic modifications regulating trained immunity are influenced by multiple factors, such as genetic background and lifestyle-related variables including the circadian rhythm, diet, physical inactivity, and psychosocial stress.^29,30^ Therefore, interindividual variability adds layers of complexity in our understanding of the specific mechanisms that regulate trained immunity, which may affect research outcomes and data interpretation.

Experimental mouse *in vitro* models provide significant advantages for investigating trained immunity. The use of inbred mice eliminates genetic variability and minimizes environmental influences, ensuring greater reproducibility and minimizing the impact of environmental factors on research outcomes. Furthermore, animals maintained under specific-pathogen-free conditions ensure the use of naive monocytes devoid of pre-existing memory. This facilitates the characterization of the training process itself, without the influence of prior exposures that may induce overlapping long-term epigenetic reprogramming.^31^ Moreover, the broad availability of congenic mouse strains has advanced our understanding of the mechanisms that regulate innate immune responses,^32^ is critical for determining genetic and environmental factors that regulate the immune system,^33,34^ and has been used to dissect gene-specific contributions during the induction of trained immunity.^35^

To establish a common framework of methodological standards that experimentally reproduce the definition of trained immunity, we developed an *in vitro* protocol using murine bone-marrow-derived macrophages, sequentially stimulated with β-glucan and LPS. Using this methodology, we demonstrate that trained macrophages significantly secrete higher levels of IL-6 and TNF following β-glucan stimulation compared to unstimulated macrophages (step 1) but do not secrete significant cytokine levels during the resting period (step 2). Upon secondary LPS stimulation, trained macrophages exhibit a rapid and enhanced production of IL-6 and TNF compared to macrophages individually stimulated with either β-glucan (step 3) or LPS (step 4). The combined cytokine production of macrophages individually stimulated with either β-glucan (stimulus 1) or LPS (stimulus 2) was significantly lower than the cytokine levels produced by trained macrophages sequentially stimulated with both β-glucan and LPS (stimulus 1 + 2) (step 5). These results experimentally reproduce the distinct functional stages that macrophages undergo during the induction of trained immunity associated with metabolic, transcriptomic, and epigenetic reprogramming. Using this methodology, we identified the serum amyloid A3 protein (SAA3) as a biomarker of trained immunity. Functionally, trained macrophages upregulate stimulatory molecules (major histocompatibility complex class II [MHC-II], CD80, CD86, OX40L, and programmed cell death ligand 1 [PDL1]), downregulate inhibitory molecules (Inducible Co-stimulator Ligand and CD206), and induce CD4^+^ and CD8^+^ T cell proliferation through a cell-contact-dependent mechanism. Additionally, our results reveal the adverse effects of cell cryopreservation and strain-specific differences in the induction of trained immunity, providing additional insights into functional training mechanisms regulated by mTOR.

Our findings support the concept of trained immunity as a unique functional program of macrophages that synergistically produce increased levels of inflammatory cytokines upon sequential stimulation with a resting period in between, in contrast to priming, where cytokine production is the result of the additive effect of two consecutive independent stimuli. This methodology addresses the need for standardized protocols to study trained immunity that facilitate cross-laboratory data integration and enhance rigor and reproducibility of the results, favoring the development of therapeutic strategies that target innate immune-memory responses in multiple immune-mediated diseases.

## RESULTS

### Kinetics of cytokine production demonstrate the functional steps of trained immunity

**Figure 1.**
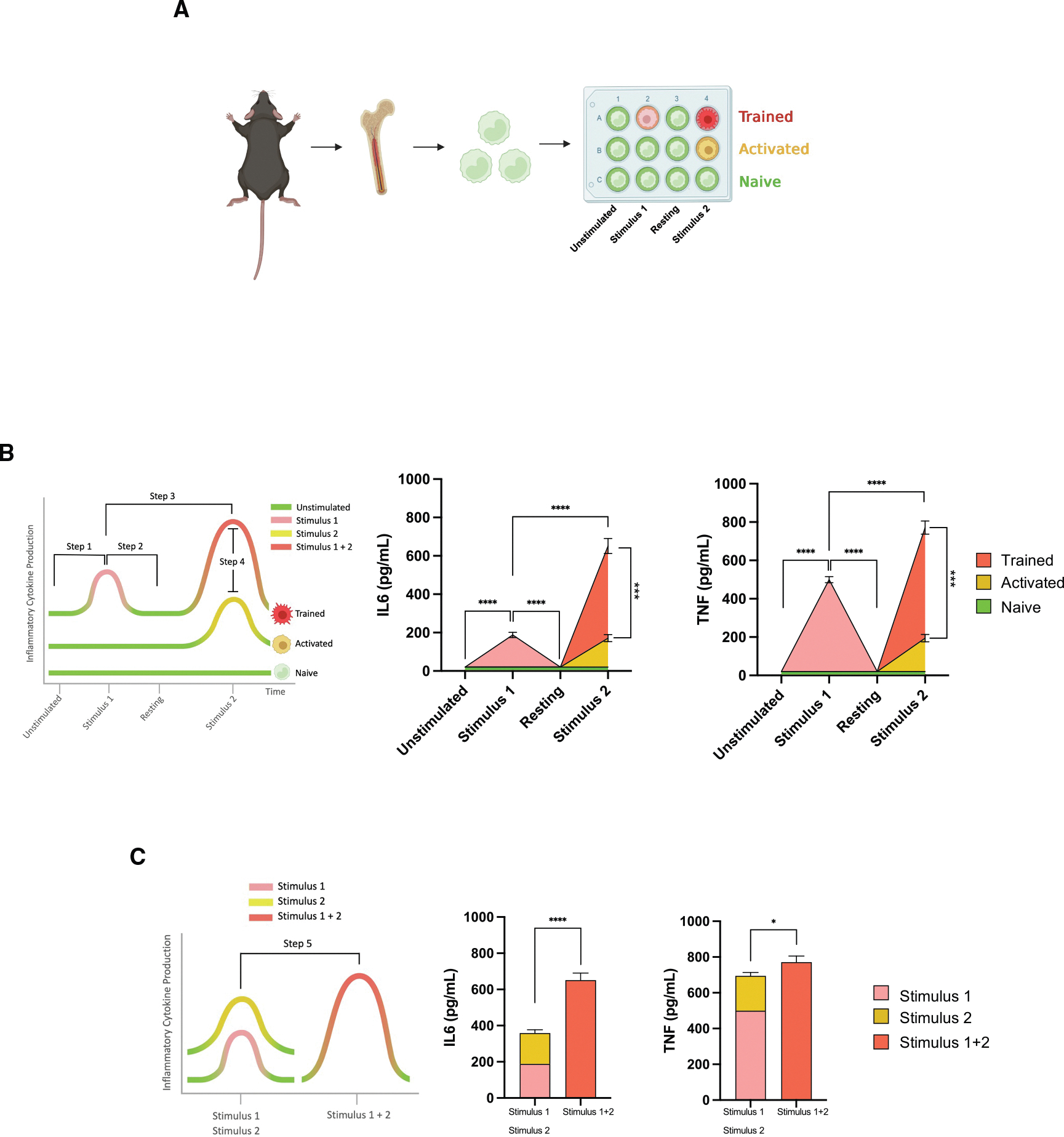
Kinetics of cytokine production demonstrate the functional steps of trained immunity (A) Schematic illustration of the *in vitro* trained-immunity protocol using bone-marrow monocytes from C57BL/6 mice. (B) Representation of four steps that define trained immunity. During the first stimulus, trained macrophages increase their functional immune status (step 1), which returns to the basal level following removal of the stimulus (step 2). In response to a secondary challenge, the function of trained macrophages is enhanced compared to macrophages after the primary (step 3) or the secondary challenge (step 4) alone. Time-course quantification of IL-6 and TNF production from naive, activated, and trained macrophages by ELISA on day 0 (unstimulated), prior to medium change on day 3 (stimulus 1), prior to LPS stimulation on day 6 (resting), and 6 h after LPS stimulation on day 6 (stimulus 2). Data are presented as mean ± SEM (*n* = 3 mice per group of three independent experiments; one-way ANOVA; **p ≤ 0.01, ***p ≤ 0.005, ****p ≤ 0.001). (C) Representation of the fifth step that defines trained immunity. The combined production of IL-6 and TNF following stimulus 1 (trained macrophages on day 3) and stimulus 2 (activated macrophages on day 6) was compared to the cytokine levels produced by trained macrophages after stimulus 1 + 2 (trained macrophages on day 6). Paired t test; *p ≤ 0.05, ****p ≤ 0.001.

A schematic overview of our methodological approach to induce trained immunity using murine bone-marrow-derived macrophages is depicted in Figure 1A. Bone-marrow monocytes were isolated by negative selection with magnetic beads (Figure S1A), as previously described,^36^ and stimulated with β-glucan in complete DMEM containing macrophage colony stimulating factor (M-CSF). After 3 days, medium was collected for cytokine analysis, and macrophages were allowed to rest for three additional days with fresh medium. On day 6, medium was collected for cytokine analysis and macrophages were re-stimulated with LPSs for 6 h. Control groups included unstimulated naive and LPS-activated macrophages. At the end of the cell-culture period, no significant differences in cell number across naive, activated, and trained macrophages were observed, with a cell viability above 95% (mean 97.0% ± 1.1% for naive, 95.9% ± 1.2% for activated, and 91.6% ± 8.4% for trained macrophages) (Figure S1B). Morphologically, trained macrophages exhibited a spherical morphology compared to naive and activated macrophages (Figure S1C), together with a significant increase in cell area (mean 193.6 ± 9.0 μm^2^ for naive, 185.9 ± 7.4 μm^2^ for activated, and 311.3 ± 12.4 μm^2^ for trained macrophages), consistent with previous reports.^8,37^

To functionally characterize naive, activated, and trained macrophages, we measured their ability to secrete pro-inflammatory cytokines at different time points. We specifically monitored the production of IL-6 and TNF before β-glucan stimulation (day 0, unstimulated), before medium was changed (day 3, stimulus 1), before LPS stimulation (day 6, resting), and after LPS stimulation (day 6 and 6 h, stimulus 2). Figure 1B shows that macrophages significantly produce increased levels of IL-6 and TNF after stimulation with β-glucan compared to unstimulated macrophages (step 1). Following a resting period, trained macrophages do not secrete significant levels of cytokines (step 2) but rapidly produce enhanced levels of IL-6 and TNF after secondary stimulation with LPS, compared to macrophages individually stimulated with either β-glucan (step 3) or LPS (step 4) (Figure 1C). The combined production of TNF and IL-6 secreted by macrophages after stimulation with either β-glucan (stimulus 1) or LPS (stimulus 2) alone was significantly lower than the cytokine levels produced by trained macrophages sequentially stimulated with both β-glucan and LPS (stimulus 1 + 2) (step 5). These results demonstrate that trained immunity is a unique functional program that mediates the synergistic production of pro-inflammatory cytokines upon secondary stimulation, in contrast to priming, where cytokine production results from the additive effects of two consecutive independent stimuli without a resting phase.

### Trained macrophages undergo metabolic reprogramming

**Figure 2.**
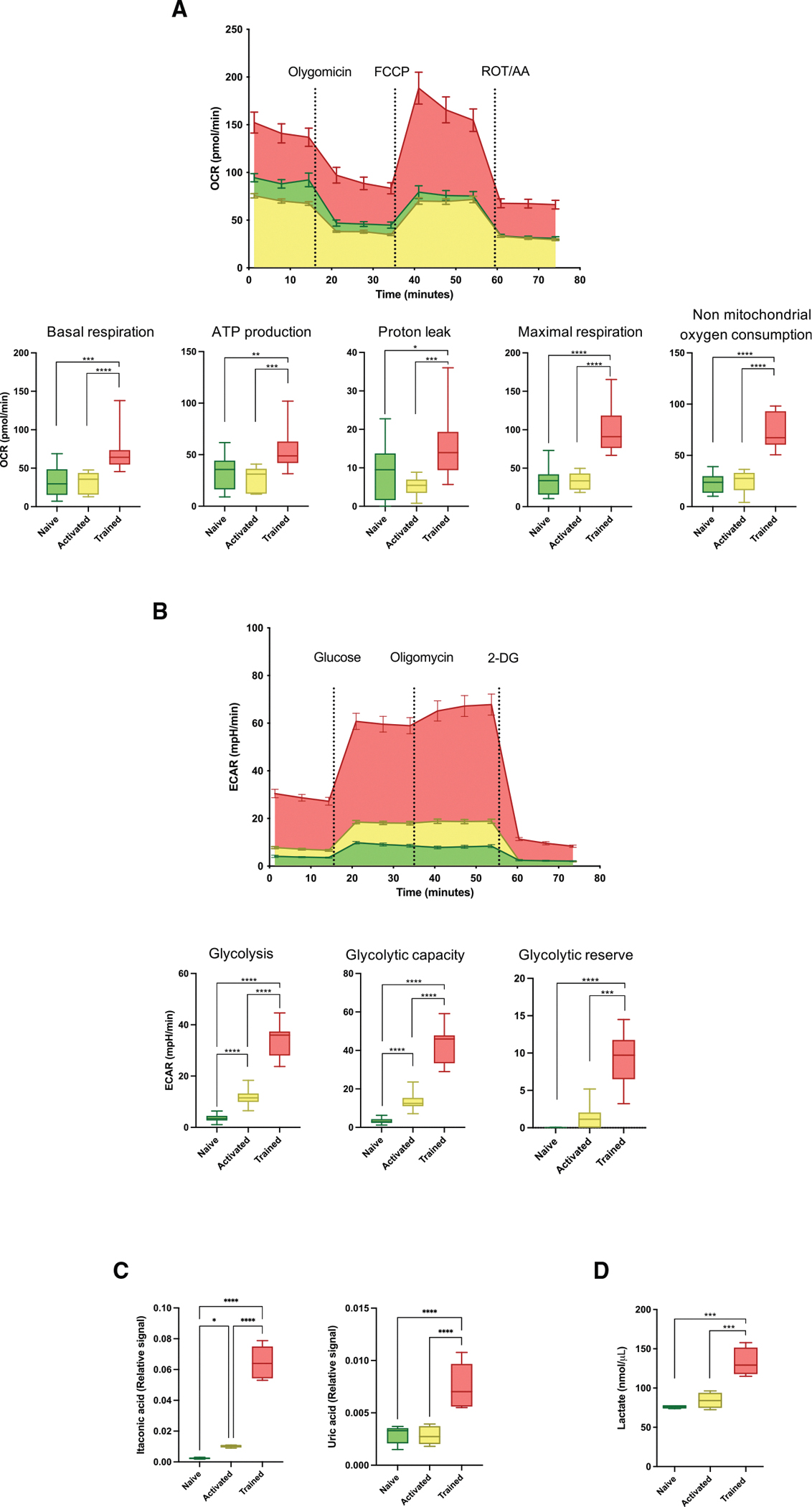
Trained macrophages undergo metabolic reprogramming (A and B) Seahorse assay analysis of (A) glycolytic metabolism and (B) mitochondrial respiration in naive, activated, and trained macrophages. Data are presented as mean ± SEM (*n* = 4 mice per group of three independent experiments; one-way ANOVA with Tukey’s multiple comparisons test; *p ≤ 0.05, **p ≤ 0.01, ***p ≤ 0.005, ****p ≤ 0.001). (C) Quantification of intracellular itaconic and uric acid by mass spectrometry analysis. Data are presented as mean ± SEM (*n* = 4 mice per group of two independent experiments; one-way ANOVA; *p ≤ 0.05, ****p ≤ 0.001). (D) Quantification of lactate production by colorimetric assay. Data are presented as mean ± SEM (*n* = 4 mice per group; one-way ANOVA; ***p ≤ 0.005).

Since β-glucan reprograms the metabolic landscape of monocytes,^38^ we next sought to characterize the metabolic profile of macrophages. To assess the mitochondrial function of macrophages, we quantified the oxygen consumption rate (OCR) during a mitochondrial stress test. Our results showed that OCR basal respiration, ATP production, and proton leak were increased in trained macrophages. OCRs were also measured by quantifying maximal respiration and non-mitochondrial oxygen consumption via oxidative phosphorylation (OXPHOS), which was significantly upregulated in trained macrophages (Figure 2A). Evaluation of the glycolytic activity revealed an increased extracellular acidification rate (ECAR) in trained macrophages from basal levels following sequential addition of glucose, oligomycin, and 2-deoxy-D-glucose (2-DG) to the medium. ECAR measurements confirmed a metabolic shift in trained macrophages with increased glycolytic activity (Figure 2B). Having characterized the oxidative phosphorylation and glycolytic properties of macrophages, we next determined their relative expression of polar metabolites by mass spectrometry. Our results revealed a significant accumulation of uric acid and itaconic acid in trained macrophages (Figure 2C), consistent with the induction of innate immune memory.^39,40^ Additionally, we measured the lactate production by macrophages, which represents a central hallmark of trained immunity,^16^ and our results confirmed that trained macrophages secrete higher levels of lactate compared to naive and activated macrophages, confirming their metabolic reprogramming (Figure 2D).

### Transcriptomic and epigenetic analyses identify SAA3 as a biomarker of training

**Figure 3.**
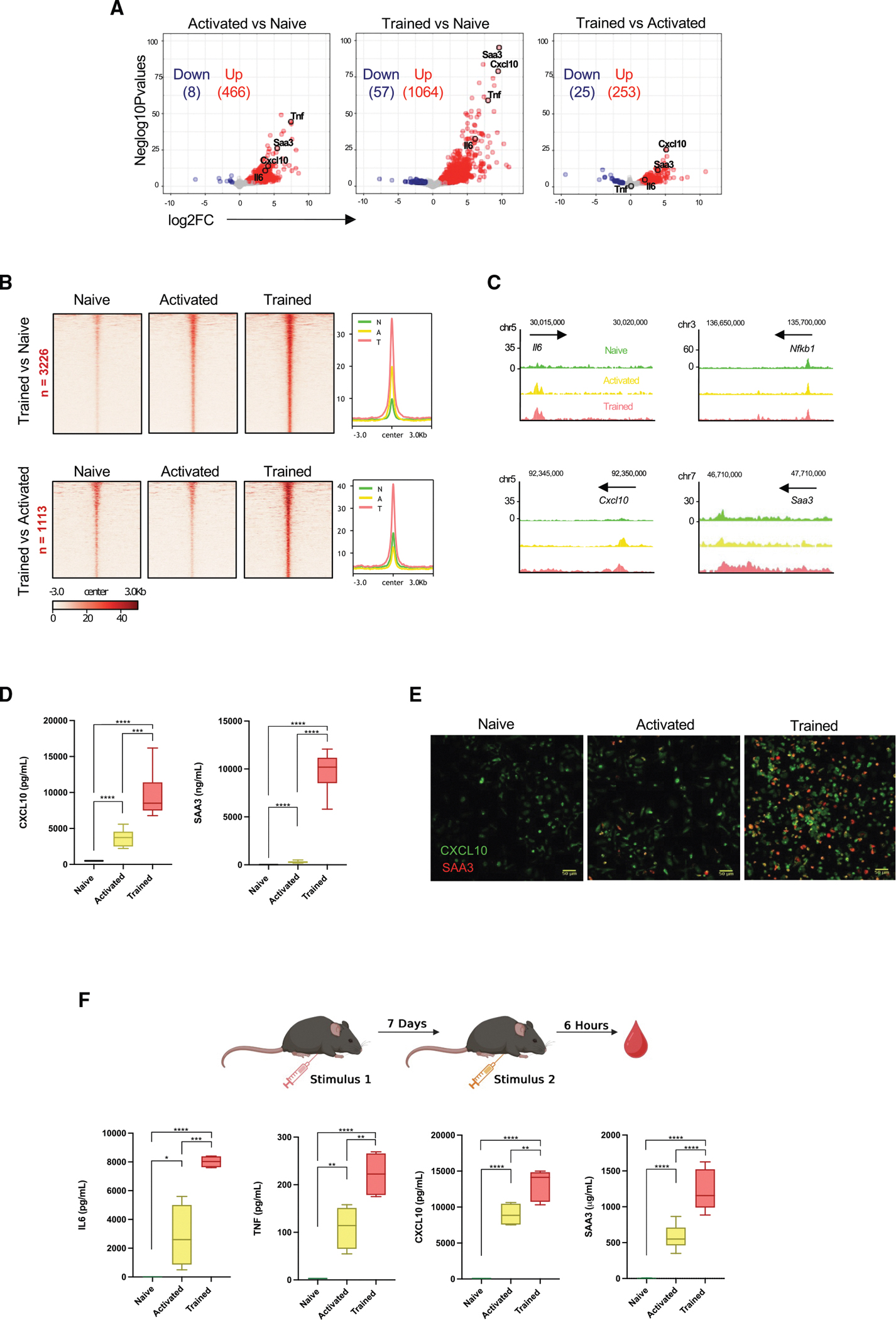
Transcriptomic and epigenetic analyses identify SAA3 as biomarker of training (A) Volcano plots of expressed genes in naive, activated, and trained macrophages. (B) ATAC-seq signal within differentially accessible regions (DARs) showing differences in the open-chromatin landscape among the three conditions. (C) ATAC-seq genome tracks of *Il6*, *NF-κB1*, *Cxcl10*, and *Saa3*. (D) Quantification of CXCL10 and SAA3 production from naive, activated, and trained macrophages after 6 h of LPS stimulation by ELISA. Data are presented as mean ± SEM (*n* = 4 mice per group of three independent experiments; one-way ANOVA; ***p ≤ 0.005, ****p ≤ 0.001). (E) Representative immunofluorescence images of CXCL10 and SAA3 in macrophages from naive, activated, and trained macrophages (*n* = 4 mice per group of two independent experiments. 5× magnification; scale bar, 50 μm). (F) Schematic illustration of the *in vivo* trained-immunity protocol. IL-6, TNF, CXCL10, and SAA3 protein quantification in the serum of naive, activated, and trained mice was measured after 6 h of LPS injection. Data are presented as mean ± SEM (*n* = 8 mice per group; one-way ANOVA with Tukey’s multiple comparisons test; *p ≤ 0.05, **p ≤ 0.01, ****p ≤ 0.001).

To characterize the transcriptional profile of naive, activated, and trained macrophages, we extracted their RNA and performed RNA-seq. Data analysis identified several differentially expressed genes (DEGs) in trained macrophages, including known pro-inflammatory genes associated with training, such as IL-6 and TNF, together with the recently reported trained-immunity marker in BCG-stimulated human monocytes, CXCL10,^41^ and the mouse-specific trained-immunity marker serum amyloid A3 protein (SAA3) (Figure 3A). Since trained immunity is characterized by epigenetic reprogramming,^42^ we next evaluated chromatin accessibility in naive, activated, and trained macrophages by Assay for Transposase-Accesible Chromatin with sequencing. The signal within ATAC peaks revealed differences in the chromatin landscape, indicating that trained macrophages presented the highest number of differentially accessible regions (DARs) (Figure 3B). Notably, trained macrophages exhibited increased accessibility of peaks near promoters of genes relevant to trained immunity, including *Cxcl10* and *Saa3* (Figure 3C). The *in vitro* production of CXCL10 and SAA3 by trained macrophages confirmed their ability to secrete enhanced levels of these proteins (Figure 3D), indicating a strong correlation between transcriptomic (RNA-seq), epigenetic (ATAC-seq), and proteomic (ELISA) results. Additional protein expression measured by immunofluorescence showed that CXCL10 and SAA3 were significantly increased during training (Figure 3E). Finally, we sought to confirm our *in vitro* findings using a previously described *in vivo* mouse model of training.^43^ Briefly, β-glucan was administered intraperitoneally (i.p.) into C57BL/6 mice, and, after 6 days of resting, they were re-stimulated with LPS i.p. Consistent with our *in vitro* data, cytokine levels of IL-6, TNF, CXCL10, and SAA3 were significantly increased in the serum of trained mice (Figure 3F). Overall, our results confirm that induction of trained immunity is driven by orchestrated epigenetic modifications that reprogram chromatin accessibility, enabling the rapid transcription of genes encoding pro-inflammatory cytokines and signaling molecules to mount faster and stronger immune responses. Altogether, the data validate our methodology as an effective *in vitro* mouse model for studying trained immunity.

### Trained macrophages upregulate signals 1, 2, and 3 and induce T cell proliferation

**Figure 4.**
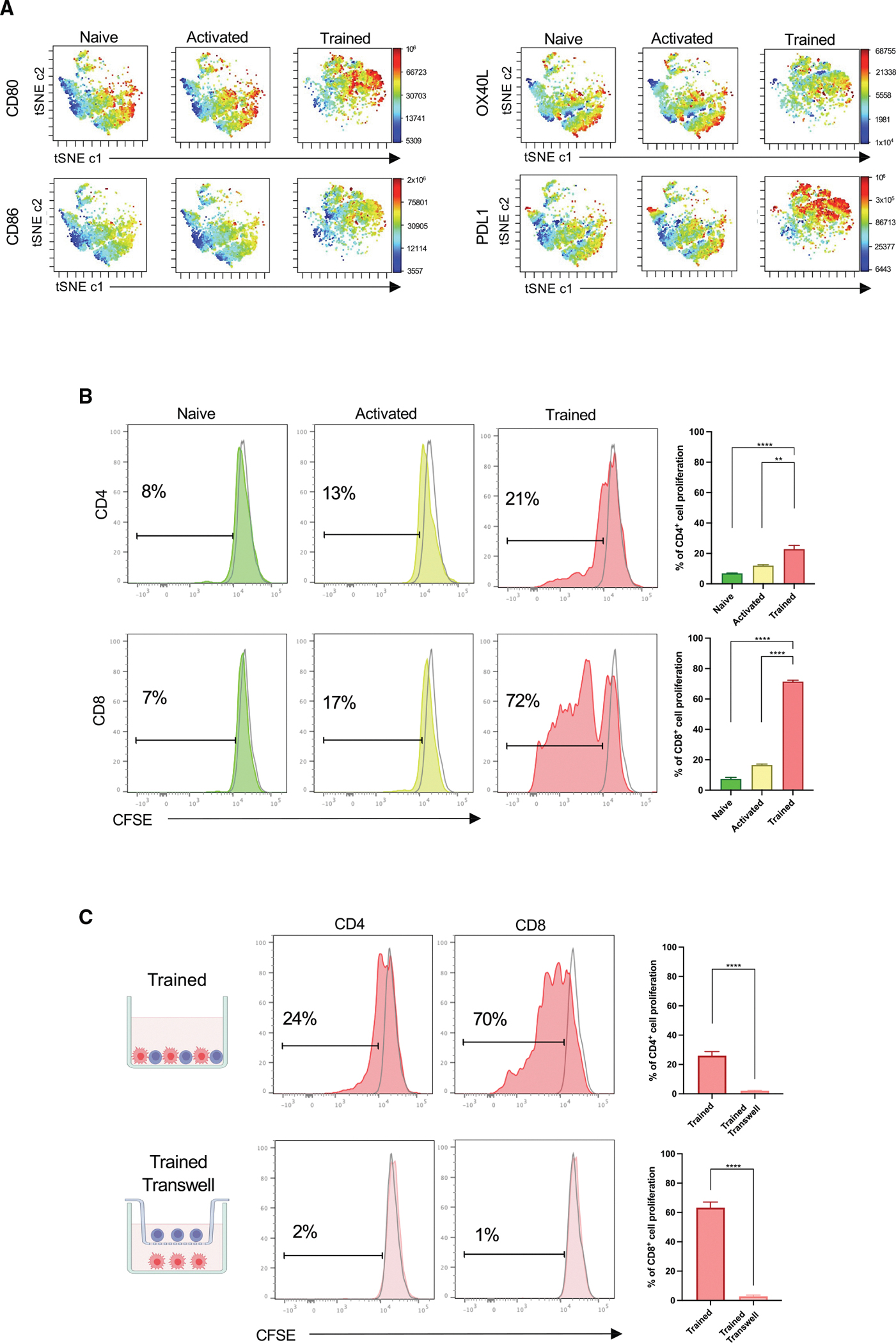
Trained macrophages upregulate signals 1, 2, and 3 and induce T cell proliferation (A) t-Distributed Stochastic Neighbor Embedding analysis of co-stimulatory molecules CD80, CD86, OX40L, and PDL1 in naive, activated, and trained macrophages measured by flow cytometry (*n* = 6 mice per group). (B) Representative histograms and quantification of proliferating CFSE-labeled CD4^+^ and CD8^+^ T cells co-cultured with naive, activated, and trained macrophages. Data are presented as mean ± SEM (*n* = 4 mice per group of three independent experiments; one-way ANOVA; **p ≤ 0.01, ***p ≤ 0.005, ****p ≤ 0.001). (C) Representative histograms and quantification of proliferating CFSE-labeled CD4^+^ and CD8^+^ T cells co-cultured with trained macrophages using a Boyden chamber (Transwell) with 0.4-μm membrane pore size. Data are presented as mean ± SEM (*n* = 4 mice of two independent experiments; paired t test; ****p ≤ 0.001).

Since trained macrophages secrete higher levels of pro-inflammatory cytokines (signal 3), we next investigated their expression of molecules involved in signals 1 and 2, which are required for successful T cell activation.^44^ High-dimensional data visualization by t-SNE analysis of the expression of co-stimulatory molecules CD80, CD86, OX40L, and PDL1 by spectral flow cytometry revealed differences in clustered peninsular locations across naive, activated, and trained macrophages (Figure 4A). Significant upregulation of these markers, together with MHC-II, were confirmed by manual gating, while the inhibitory CD206 and ICOSL molecules were downregulated in trained macrophages (Figure S2). The upregulation of antigen presentation and co-stimulatory molecules (signals 1 and 2), combined with enhanced cytokine production (signal 3), suggests that trained macrophages are fully equipped to activate T cells *in vitro*. We next co-cultured CFSE-labeled splenic CD4^+^ and CD8^+^ T cells with macrophages, as previously described,^45^ and our results show strong T cell proliferation rates in trained (CD4^+^ 26.6% ± 9.2%, CD8^+^ 72.6% ± 8.1) compared to naive (CD4^+^ 12.8% ± 6.3%, CD8^+^ 18.4% ± 9.4%) and activated (CD4^+^ 13.9% ± 7.1%, CD8^+^ 20.1% ± 4.9%) macrophages (Figure 4B). We also determined whether trained macrophages induced T cell proliferation in a cell-contact-dependent manner using a Boyden chamber, where CFSE-labeled T cells were placed on the upper layer of the cell-culture insert with a permeable membrane, while macrophages were placed at the bottom. Remarkably, CD4^+^ and CD8^+^ T cell proliferation was significantly decreased in this assay, indicating that direct contact between trained macrophages and T cells is necessary for CD4^+^ and CD8^+^ T cells to proliferate *in vitro* (Figure 4C).

### Effects of cryopreservation and genetic ablation on trained macrophages

**Figure 5.**
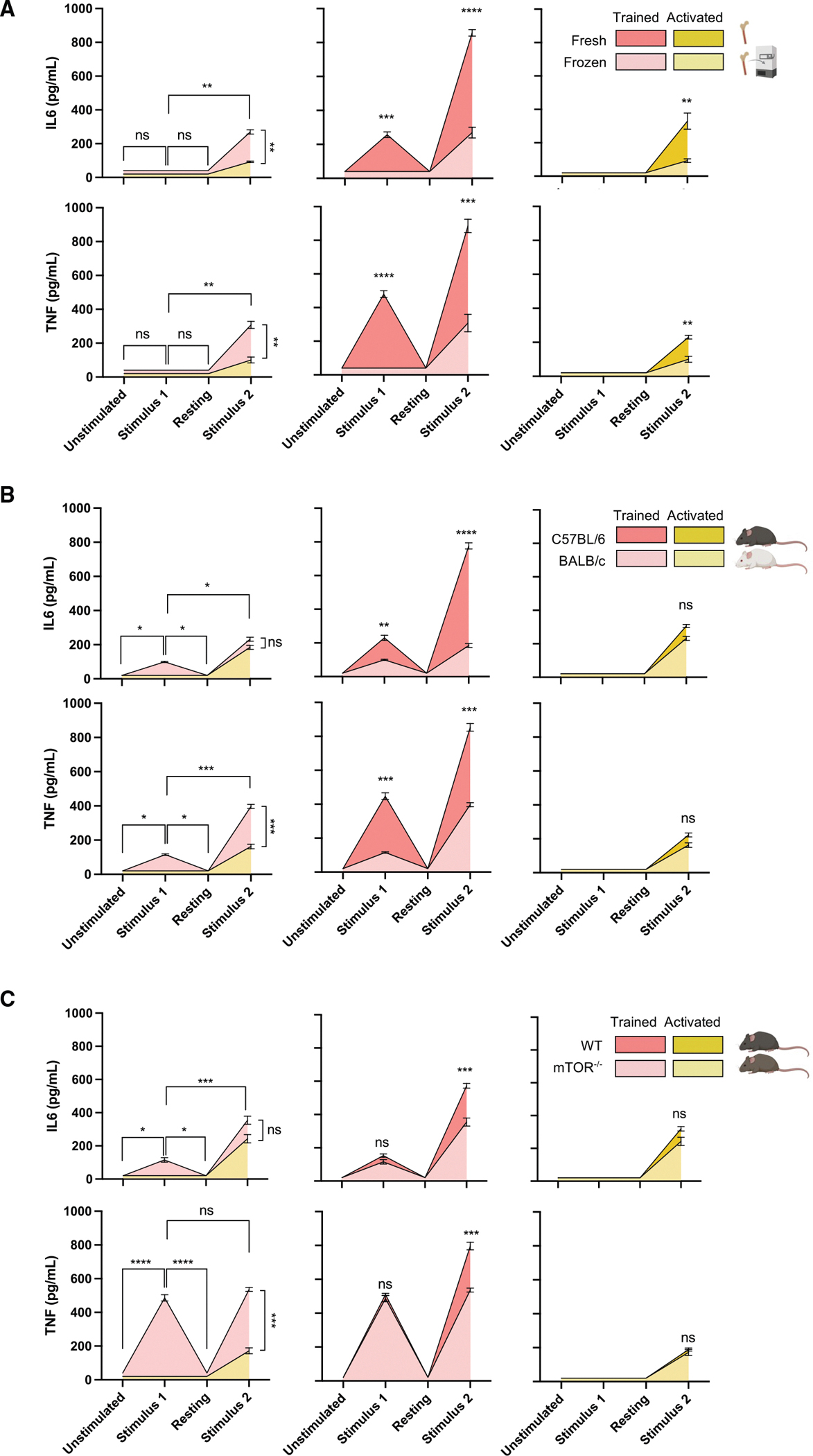
Effects of sample cryopreservation and mouse genetics on the induction of training Time-course quantification of IL-6 and TNF production by macrophages (A) differentiated from freshly isolated or cryopreserved monocytes, (B) from C57BL/6 or BALB/C mice, and (C) from wild-type or mTOR-deficient mice. Data are presented as mean ± SEM (*n* = 3 mice per group of three independent experiments; one-way ANOVA or paired t test; **p ≤ 0.01, ***p ≤ 0.005, ****p ≤ 0.001).

Sample processing, cryopreservation, and transportation of bone-marrow cells are routinely performed in multicenter collaborative research projects and clinical trials.^46^ To evaluate the effects of cryopreservation on trained immunity, we cryopreserved freshly isolated bone marrow in liquid nitrogen for 24 h. After thawing, monocytes were isolated by negative selection with magnetic beads and cultured side by side with freshly isolated monocytes to generate macrophage subsets. Kinetics of IL-6 and TNF production revealed that trained macrophages derived from cryopreserved cells did not secrete significant levels of IL-6 and TNF following stimulus 1 (step 1) (Figure 5A). Interestingly, significant differences between trained and activated macrophages were observed at step 4, emphasizing the importance of longitudinal cytokine measurements to assess training. Additionally, cryopreserved monocytes differentiated into macrophages displayed impaired IL-6 and TNF production following LPS stimulation, suggesting that both activation and training of macrophages may be conditioned by cryopreservation.

C57BL/6 and BALB/c are the most widely used mouse strains for studying the immune response. To investigate whether trained immunity differs between these strains, we compared the kinetics of IL-6 and TNF production by bone-marrow monocytes from C57BL/6 and BALB/c mice differentiated into trained macrophages (Figure 5B). The results showed that trained macrophages from BALB/c mice secreted IL-6 levels comparable to those of LPS-activated macrophages (step 4). However, when comparing IL-6 and TNF kinetics between strains, trained macrophages from BALB/c mice produced lower levels of both cytokines following stimuli 1 and 2. In contrast, macrophages from both strains secreted similar levels of IL-6 and TNF after LPS stimulation, suggesting comparable activation but different training capacities between macrophages derived from C57BL/6 and BALB/c mice.

C57BL/6 is the most commonly used inbred mouse strain in experimental research due to the availability of congenic models with genetic mutations, enabling detailed studies on the function of specific genes. Previous studies demonstrated that the mTOR plays a central role in the production of IL-6 and TNF by human monocytes trained with sequential stimulation with β-glucan and LPS.^17,47^ To further characterize the role of mTOR in this process, we examined the kinetics of IL-6 and TNF production by trained macrophages derived from mTOR-deficient monocytes. Unexpectedly, our results show an increased production of IL-6 and TNF following stimulus 1 (step 1) (Figure 5C), although it was not enhanced after LPS stimulation. While TNF production was not increased between stimuli 1 and 2 (step 3), IL-6 production was not increased between activated and trained macrophages after stimulus 2 (step 4). These findings indicate that mTOR deficiency does not affect macrophage cytokine production following individual stimulation with either β-glucan or LPS alone but limits their ability to produce enhanced levels of IL-6 and TNF after secondary stimulation. These results underscore the importance of longitudinally assessing IL-6 and TNF production by trained macrophages to accurately characterize their functionality.

## DISCUSSION

There is an urgent need for standardized protocols that study trained immunity to promote data reproducibility and integration of findings across different laboratories. Experimental *in vitro* mouse models represent a largely unexplored methodological approach for studying trained immunity, offering synergistic benefits to existing protocols. Mice are maintained in specific-pathogen-free environments under controlled conditions of diet, stress, and light cycles, which significantly decrease the impact of confounding factors that may introduce variability in the study of innate immune-memory processes. Therefore, mouse models guarantee the use of naive monocytes without pre-existing memory for sequential *in vitro* stimulation with trained-immunity-inducing agents. In addition, the use of inbred mice facilitates data reproducibility in research studies by providing genetic uniformity that accelerates the discovery and validation of biomarkers by enabling targeted gene modifications.^33^ Unfortunately, *in vitro* protocols for studying trained immunity using murine peripheral blood monocytes remain undeveloped due to the limited number of circulating monocytes, which are insufficient for comprehensive phenotypic, functional, metabolic, transcriptomic, and epigenetic assays.^48^ Recently, the bone marrow has emerged as a valid source of cells for trained-immunity studies,^49^ based on previous *in vivo* studies demonstrating β-glucan-dependent long-term epigenetic reprogramming in hematopoietic progenitors.^25^ However, methodological differences of bone-marrow-derived monocyte isolation and differentiation protocols influence the metabolic and epigenetic reprogramming of monocytes associated with the secretion of effector molecules.^50,51^ As a result, integration of trained-immunity findings reported by different laboratories obtained using different methodological approaches remains a challenging task to avoid confusion in the literature and encourage progress in this field.

To advance our understanding of the underlying mechanisms that regulate trained immunity, we developed an *in vitro* methodology that uses murine bone-marrow monocytes under standard cell-culture conditions. Since the induction of trained immunity is functionally assessed by increased pro-inflammatory cytokine secretion, we first evaluated the ability of bone-marrow-derived macrophages to produce enhanced levels of IL-6 and TNF following sequential stimulation with β-glucan and LPS. Kinetics of cytokine production revealed that, following stimulation with β-glucan, trained macrophages secrete higher levels of inflammatory cytokines compared to unstimulated naive macrophages. Cytokine secretion returned to basal levels during a resting period but was significantly enhanced upon secondary stimulation with LPS compared to macrophages stimulated with either β-glucan or LPS alone. The combined production of IL-6 and TNF individually secreted by trained macrophages after stimulus 1 (day 3) and activated macrophages after stimulus 2 (day 6) was significantly lower than the levels of IL-6 and TNF produced by trained macrophages after stimulus 1 and 2 (day 6). These experimentally reproduce the functional defining steps that characterize trained immunity.

Differential analysis of RNA-seq and ATAC-seq data identified serum amyloid A3 protein (SAA3) as a potential biomarker of trained immunity. While *SAA3* is a pseudogene in humans,^52^ the murine *Saa3* gene encodes a functional acute-phase protein that is induced under inflammatory conditions and is regulated by IL-1, IL-6, and TNF.^53–55^ SAA3 acts as a functional ligand for TLR4 in macrophages and, following LPS stimulation, SAA3 further induces nuclear factor (NF)-κB signaling and upregulation of IL-6 and TNF production.^56–59^ This suggests that SAA3 secretion by trained macrophages may be part of a feedback mechanism to sustain longer and protective inflammatory cytokine production after LPS stimulation, which may explain the increased levels of IL-6 and TNF in trained macrophage cell cultures. Consistent with these findings, previous *in vivo* studies demonstrated the protective role of SAA3 in bacterial infections via macrophage-derived IL-1β production.^60,61^ Therefore, it is possible that early SAA3 secretion by trained macrophages may be important to control the acute phase of bacterial infections.^62,63^ Although SAA3 is not present in humans, its identification as a trained-immunity-related protein highlights the potential clinical relevance of SAA family proteins as biomarkers in immune-related diseases.^64–66^

Phenotypic characterization of trained macrophages revealed upregulation of stimulatory molecules (MHC-II, CD80, CD86, PDL1, and OX40L) and downregulation of inhibitory molecules (ICOSL and CD206). Consistent with this phenotype, *in vivo* BCG training has been shown to increase MHC-II and CD80 expression in murine macrophages.^67^ Although the functional role of OX40L in trained macrophages has not been studied in detail, previous studies demonstrated that OX40L stimulation induces coordinated upregulation of pro-inflammatory genes (*Tnf*, *Il6*, and *Cxcl10*) and trained-immunity-associated transcription factors (*Hif1a*, *Nos2*).^68^ Notably, molecular interactions between OX40L-expressing macrophages and OX40-expressing CD4^+^ and CD8^+^ T cells provide co-stimulatory signals that enhance T cell activation, cytokine production, proliferation, and survival.^69,70^ Similarly, PDL1-expressing macrophages have been reported to stimulate T cells and are associated with improved survival outcomes in breast cancer patients.^71^ The functionality of PDL1-expressing macrophages appeared to be independent of PDL1-programmed cell death protein 1 (PD1) interactions, suggesting that combining anti-PDL1 therapy with induction of PDL1 expression in macrophages have synergistic therapeutic benefits. Consistent with this hypothesis, simultaneous inhibition of PDL1-PD1 interactions and induction of trained immunity has been shown to elicit durable anti-tumor responses that significantly reduce tumor growth in murine melanoma models.^72^ On the other hand, macrophages expressing ICOSL and CD206 are associated with anti-inflammatory responses and have been reported to produce lower levels of IL-6 and TNF following LPS stimulation.^73,74^ The observed downregulation of these inhibitory molecules in trained macrophages is consistent with an enhanced stimulatory immune phenotype.

T cell activation requires stimulation through MHC (signal 1), co-stimulation (signal 2), and inflammatory cytokines (signal 3).^75^ Since trained macrophages upregulate signals 1, 2, and 3, we explored their ability to induce CD4^+^ and CD8^+^ T cell proliferation *in vitro*. Our findings demonstrate that trained macrophages promote T cell proliferation in a cell-contact-dependent manner. To the best of our knowledge, this is the first study that reports the ability of trained macrophages to induce T cell proliferation. Prior studies have highlighted the role of T cell-derived interferon gamma (IFNγ) in macrophage training, as alveolar macrophages require direct CD8^+^ T cell interaction and IFNγ signaling for trained-immunity induction following adenoviral vector vaccination and secondary challenge by *Streptococcus pneumoniae*.^76^ Moreover, IFNγ-producing BCG-specific CD4^+^ T cells mediate heterologous viral protection against SARS-CoV-2 and PR8 influenza by imprinting an antiviral program in lung myeloid cells.^77^ Additional studies demonstrated that IFNγ-producing BCG-specific CD4^+^ T cells confer heterologous viral protection against SARS-CoV-2 and PR8 influenza by imprinting a persistent antiviral program in lung myeloid cells.^78^ The necessity of T cell-derived IFNγ and other soluble factors in monocyte training was further confirmed using PBMCs, where depletion of T cells abrogated upregulation of IL-6 and TNF after training with *Plasmodium falciparum*. More recently, heat-killed *C. albicans* (HKCA) was shown to increase IL-6 and TNF secretion in PBMCs after LPS stimulation, supporting that monocyte-T cell interactions are necessary for optimal IFNγ production and effective induction of trained immunity. While these studies highlight the importance of T cell-derived IFNγ in monocyte training, other evidence supports T cell-independent mechanisms,^8,12,35^ suggesting that variations in monocyte isolation and culture conditions may account for the discrepancies in these findings.

We further compared the training capacity of monocytes using two different mouse strains. Unexpectedly, we observed that trained macrophages from BALB/c mice produced lower levels of IL-6 and TNF than those from C57BL/6 mice, suggesting that trained immunity is conditioned by genetic background. Previous studies have shown that LPS-stimulated macrophages from BALB/c mice exhibit a delayed gene regulation compared to C57BL/6 macrophages, affecting cell signaling, cytokine expression, oxidative stress, and lipid metabolism, which ultimately impacts macrophage activation.^79^ Consistently, macrophages from C57BL/6 mice have been reported to secrete higher levels of TNF than BALB/c mice after LPS stimulation *in vitro*.^80^ Further comparisons revealed that strain-specific differences in the respiratory-chain complexes, lysosomal enzymes, and antioxidant-stress systems provide advantages to control microbial pathogens in macrophages generated from C57BL/6 compared to BALB/c mice.^81^ Strain-specific resistance to infectious agents may be partially explained by differences in pathogen phagocytosis and inflammatory signaling,^82^ since β-glucan-mediated induction of trained immunity requires phagocytosis and subsequent lysosomal mTOR activation for metabolic and epigenetic reprogramming.^83^ Our results align with a recent *in vivo* study demonstrating that trained C57BL/6 mice developed stronger antibacterial immunity against a secondary infection with *Streptococcus pneumoniae* compared to BALB/c mice.^76^ Since β-glucan induces trained immunity in human monocytes via the mTOR pathway,^17^ we trained mTOR-deficient bone-marrow monocytes to confirm these results using congenic mice. Our *in vitro* results align with previous findings that used rapamycin for pharmacological inhibition, confirming that mTOR is required for enhanced cytokine secretion after secondary stimulation. Unexpectedly, cytokine production was not affected after β-glucan stimulation (stimulus 1), and mTOR-deficient monocytes secreted levels of IL-6 and TNF comparable to wild-type monocytes. These results suggest that mTOR may be essential to either induce and/or maintain long-term epigenetic reprogramming and chromatin accessibility associated with enhanced cytokine production upon secondary challenge. These findings highlight the value of genetically modified inbred mouse strains to study trained immunity and underscore the importance of *in vitro* methodologies to characterize the dynamics of training over time.

Our findings also have significant implications for the use of cryopreserved PBMCs in clinical studies, where sample collection at different time points requires extended sample storage under cryopreservation conditions. Cryopreservation affects PBMC and monocyte functionality, reducing IL-6 and TNF secretion following LPS stimulation.^84,85^ Notably, this effect is not observed following monocyte culture and differentiation *in vitro*, since freshly isolated and thawed monocytes cultured for 7 days exhibit comparable IL-6 and TNF transcript levels after LPS stimulation.^86^ Our data revealed that macrophages derived from cryopreserved murine monocytes exhibit impaired IL-6 and TNF production after LPS stimulation compared to macrophages derived from freshly isolated monocytes. These findings highlight the need to consider cryopreservation effects when designing experiments involving trained immunity and inter-laboratory resource sharing. These findings also have significant implications for the implementation of the monocyte activation test (MAT) as an alternative to the rabbit pyrogen test, which currently requires approximately 400,000 rabbits annually worldwide.^87^ Understanding the functional properties of cryo-preserved monocytes is essential for transitioning toward alternatives to live-animal use.

Methodological differences in monocyte isolation and culture conditions significantly influence functional outcomes. Monocyte isolation by density-gradient centrifugation and plastic adhesion results in low purity, reduced viability, and activation of pro-inflammatory signaling pathways.^88^ Alternatively, positive selection of monocytes targeting CD14 may interfere with IL-6 and TNF production after LPS stimulation,^89^ likely due to TLR4 internalization following anti-CD14 antibody binding.^90^ Conversely, negative selection preserves monocyte viability and maintains their ability to secrete TNF in response to LPS stimulation.^91^ Overall, these findings highlight the benefits of monocyte negative selection offers to preserve cell viability and functionality for downstream research studies. Besides distinct monocyte isolation options, different stimulatory factors may be used for *in vitro* differentiation and survival. Macrophage colony-stimulating factor (M-CSF) and granulocyte-macrophage colony-stimulating factor (GM-CSF) are the two predominant growth factors used for monocyte cell cultures, but they distinctly influence cell phenotype and function.^92^ GM-CSF has previously been reported to induce trained immunity in both human and mouse monocytes differentiated *in vitro*. Human monocytes differentiated with GM-CSF, but not M-CSF, produce enhanced levels of TNF after LPS stimulation,^93^ suggesting that GM-CSF may act as the first stimulus during the induction of trained immunity. Consistent with these findings, Lin^−^ murine bone-marrow monocytes differentiated with GM-CSF, but not M-CSF, produce higher levels of IL-6 and TNF after LPS stimulation.^94^ As an alternative to recombinant M-CSF, L929 fibroblast-derived supernatant has been used to differentiate bone-marrow monocytes in trained-immunity studies.^95^ L929 supernatant contains over 2,000 proteins that modulate innate immune responses by enhancing glucose metabolism and mitochondrial reactive oxygen species (ROS) production, influencing the secretion of IL-6 and TNF in macrophages following LPS stimulation.^96,97^ These results highlight the impact of growth factors on the reproducibility of trained-immunity results using *in vitro* differentiated bone-marrow monocytes.

Given the wide range of protocols used for *in vitro* trained-immunity studies, including variations in monocyte isolation (density gradient, positive/negative selection, cell sorting), culture media (RPMI or DMEM), differentiation factors (M-CSF, GM-CSF, or L929 supernatant), and training stimuli (β-glucan or HKCA), at least 60 different methodologies have been reported to study trained immunity *in vitro* using mouse cells. Here, we have developed a robust *in vitro* methodology to consistently induce trained immunity, providing a valuable resource for identifying previously unrecognized biomarkers and elucidating mechanistic pathways involved in innate immune-memory responses, with potential therapeutic applications.^98^

### Limitations of the study

This resource article aims to establish a common framework for studying trained immunity in mice using bone-marrow-derived macrophages. Our results successfully reproduce the five functional steps that define trained immunity, as outlined in the consensual definition proposed in 2021 programs.^21^ However, our experiments primarily focused on the functional response of trained macrophages by measuring longitudinal IL-6 and TNF cytokine production at four distinct time points. In contrast, transcriptomic and epigenetic analyses were only conducted at a single time point—following secondary stimulation. Future studies examining transcriptomic and epigenetic changes across multiple time points are needed to fully capture the dynamic nature of training. Finally, although SAA3 was identified as a specific marker of training in mice, it is a pseudogene in humans. Nonetheless, this finding highlights the potential relevance of other SAA family members in human models of trained immunity.

### RESOURCE AVAILABILITY

#### Lead contact

Further information and requests for resources and reagents should be directed to and will be fulfilled by the lead contact, Jordi Ochando (jordi.ochando@mssm.edu).

#### Materials availability

This study did not generate new unique reagents.

#### Data and code availability

- Mass spectrometry metabolomics data have been deposited in the National Metabolomics Data Repository (NMDR)^99^ and can be accessed at http://doi.org/10.21228/M8583B.
- The project ID for the mass spectrometry metabolomics data is PR002549.
- The project DOI for the mass spectrometry metabolomics data is http://doi.org/10.21228/M8583B.
- RNA-seq and ATAC-seq data have been deposited in the NCBI Gene Expression Omnibus.^25^
- RNA-seq accession number is GSE290033.
- ATAC-seq accession number is GSE290032.
- Any additional information required to reanalyze the data reported in this work is available from the lead contact upon request.

## STAR★METHODS

Detailed methods are provided in the online version of this paper and include the following:

### EXPERIMENTAL MODEL AND STUDY PARTICIPANT DETAILS

#### Animals

The wild-type C57BL/6 mouse (000664), the BALB/cJ (000651), the B6.129P2-Lyz2tm1(cre)Ifo/J (004781) and the B6.129S4-Mtortm1.2Koz/J (011009) were purchased from the Jackson Laboratory, Bar Harbor, ME. All mice were fed under SPF (specific pathogen free) conditions in the Icahn School of Medicine at Mount Sinai and the Instituto de Salud Carlos III. Experimental groups contained equal numbers of male and female mice. Same-sex (male/female) mice aged 8–12 week old were randomly assigned to the experimental group. The design and conduct of the animal experiments on mice were approved by the Institutional Animal Care and Use Committees (IACUC) of the Icahn School of Medicine at Mount Sinai and the Instituto de Salud Carlos III, and were carried out in strict accordance with the good veterinary practices and regulatory standards. No influence of sex were observed on the results of the study.

#### Monocyte isolation

Murine bone marrow was collected and processed as previously described.^36^ Bone marrow monocytes were isolated by negative selection using the EasySep mouse monocyte isolation kit (StemCell Technologies, Vancouver, Canada) according to the manufacturer’s instructions. Purity of monocytes was determined as percentage of live CD45^+^CD11b^+^Ly6G^−^ cells by flow cytometry.

#### Cell culture

Isolated monocytes were seeded at a concentration of 5 × 10^5^ monocytes/mL (1 × 10^5^ monocytes in 200μL of media) in 96-well plates (Corning Inc., Corning, NY). Cell culture media for all conditions was Dulbecco’s Modified Eagle Medium (DMEM) supplemented with 10% Fetal Bovine Serum (FBS), 1% Penicillin/Streptomycin (P/S), 1% HEPES, 1% sodium pyruvate, 1% MEM non-essential amino acids (Thermo Fisher Scientific, Waltham, MA), 0.001% 2-mercaptoethanol (Sigma-Aldrich, St. Louis, MO), and 30 ng/mL recombinant murine M-CSF (PeproTech, Cranbury, NJ). For cryopreservation experiments, bone marrow cells were preserved in FBS containing 10% dimethyl sulfoxide (Sigma-Aldrich) and stored in liquid nitrogen for 24h.

For naive macrophages, monocytes were cultured with media for 6 days, changing the media on day 3. For activated macrophages, monocytes were cultured with media for 6 days, changing the media on day 3, and macrophages were stimulated with 10 ng/mL of LPS from *Salmonella enterica* serotype abortus equi (Sigma-Aldrich) on day 6 for 6h. For trained macrophages, monocytes were cultured on day 0 with 10 μg/mL of β-glucan from *Saccharomyces cerevisiae* (InvivoGen, San Diego, CA) or *Candida albicans* as previously described.^38^ On day 3, the media was changed and, after 3 days of resting, trained macrophages were re-stimulated with LPS for 6h. Supernatants were collected on day 0 (unstimulated), day 3 before washing (stimulus 1), day 6 before washing (resting) and 6h after 10 ng/mL of LPS stimulation (stimulus 2) and stored at −80°C, while macrophages were recovered using a cell scraper (Corning Inc., Corning, NY) and processed for downstream applications.

#### Cell number and viability analysis

After completion of the cell culture protocol (day 6 and 6h), macrophages in 96-well plates were washed directly with pre-warmed PBS. Hoechst 33342 nuclear stain (Thermo Fisher Scientific) was added to the wells at a final concentration of 2 μg/mL in PBS at 37°C for 30 minutes. macrophages were washed with pre-warmed PBS and stained with propidium iodide (Thermo Fisher Scientific) at a concentration of 5 μg/mL in PBS. Cell number and viability analysis were performed on the Cytell^™^ Cell Imaging System (GE Healthcare, Life Sciences) using the Cell Viability BioApp.

#### In vivo animal studies

Training *in vivo* was induced as previously described.^43^ Briefly, C57BL/6 mice were trained with 45 μg/g of β-glucan from *Euglena gracilis* (Sigma-Aldrich) on day 0 intraperitoneally (i.p.) and with 1,6 μg/g of LPS from Escherichia coli (Sigma-Aldrich) on day 7 for 6h. As control groups, mice received PBS i.p. on days 0 and 7 (naive mice) or PBS i.p. on day 0 and LPS on day 7 (activated mice). After 6h, serum samples were collected and preserved at −80°C for further quantification of cytokines.

### METHOD DETAILS

#### Quantification of cytokine and lactate production

ELISA assays were used for quantification of TNF, IL6 (Thermo Fisher Scientific), CXCL10 (R&D Systems, Minneapolis, MN), and SAA3 (Merck Millipore, Burlington, MA) in cell culture supernatants and serum samples, according to the manufacturer’s instructions. Production of lactate by macrophages was measured in cell culture supernatants using the BioVision assay kit (Abcam, Cambridge, UK).

#### Seahorse metabolic assays

Energetic metabolism of naive, activated, and trained macrophages was studied using a Seahorse XF96 extracellular flux analyzer (Agilent Technologies, Santa Clara, CA). Cells were seeded in a Seahorse 96-well microplates, cultured under experimental conditions described above and, after LPS stimulation, mitochondrial and glycolysis stress tests were performed using Seahorse XF Cell Mito Stress Test kit and Seahorse XF Glycolysis Stress Test kit (Agilent Technologies). Briefly, oxygen consumption rate (OCR) was measured at baseline and after the addition of 1 mM oligomycin, 1 mM FCCP, and 0.5 mM antimycin A and rotenone (all from Agilent Technologies). Extracellular acidification rate (ECAR) was measured at baseline and after addition of 10 mM glucose (Sigma-Aldrich), 1 mM oligomycin (Agilent Technologies), and 50 mM 2-deoxyglucose (2-DG; Sigma-Aldrich)

#### Metabolomic Profiling

Metabolomic profiling of naive, activated, and trained macrophages was performed by liquid chromatographic-triple quadrupole tandem mass spectrometry (LC-QqQ-MS/MS). After culture, macrophages were resuspended in 60 μL of methanol, ultrasonicated using a UP 200S device equipped with a S2 probe (Hielscher Ultrasonics GmbH, Teltow, Germany). Organic and aqueous fractions were separated by centrifugation and analyzed in an Agilent 6470 triple quadrupole LC/MS system, using an InfinityLab Poroshell 120 HILIC-Z column (2.1×150mm, 2.7μm) (Agilent Technologies). Samples were eluted using a gradient of water in acetonitrile containing 10mM ammonium acetate and MS/MS results were obtained using the Agilent 6470 triple quadrupole system (Agilent Technologies).

#### RNA extraction and sample sequencing

Naive, activated, and trained macrophages were collected and processed for RNA isolation using the RNeasy Mini Kit (Qiagen, Hilden, Germany). Barcoded RNA-seq libraries were generated using the NEBNext^®^ Single Cell/Low Input RNA Library Prep Kit (New England Biolabs, Ipswich, MA). Size and concentration of the libraries were checked using the 2100 Bioanalyzer DNA HS chip (Agilent Technologies). Libraries with different barcodes were equimolarly pooled and concentration of each pool assessed by a Qubit^®^ fluorometer (Thermo Fisher Scientific). Sequencing was performed on the Illumina HiSeq 4000 platform (Illumina, San Diego, CA), in paired-end mode and a 100 bp of sequencing read length, yielding a minimum of 60 million quality reads per sample. FastQ files were obtained using CASAVA v1.8 software (Illumina).

#### ATAC nuclei isolation and sample sequencing

Naive, activated, and trained macrophages were collected and 5×10^4^ viable macrophages were incubated with a lysing buffer containing 10 mM PIPES pH 6.8, 100 mM NaCl, 300 mM sucrose, 3 mM MgCl2, 0.1% TritonX-100 at 4°C for 5 min. After centrifugation, supernatant was discarded, nuclei resuspended in transposase reaction buffer containing transposase Tn5 (1μL Tn5 per 5×10^4^ cells), maintained at 37°C for 30 min, followed by incubation with 0.1% SDS at 40°C for 30 min to stop the reaction. DNA was isolated with KAPA Pure beads (SPRI beads) and sample concentration assessed by a Qubit^®^ fluorometer (Thermo Fisher Scientific). For DNA library generation, qPCR with i5 and i7 primers was performed (NEXTERA barcoding). Amplified DNA was incubated with 0.5x concentration of SPRI beads, which were discarded, and supernatant was incubated with 1.8x concentration of SPRI beads. Beads were washed twice with 70% ethanol, eluted with EB, and measured with a Qubit^®^ fluorometer (Thermo Fisher Scientific). Finally, library sizes and percentages were assessed using 2100 Bioanalyzer HS (Agilent Technologies). Libraries were sequenced on the Illumina HiSeq 4000 platform (Illumina) in paired-end and 50 bp length, yielding 60 million reads/sample. Sequencing output were processed with RTA v1.18.66.3 and FastQ files for each sample were obtained using bcl2fastq v2.20.0.422 software (Illumina).

#### Microscopy

A Leica DMI3000B inverted microscope (Leica Microsystems, Wetzlar, Germany) was used to monitor changes in morphology and confluency during culture of macrophages. Cell area analysis of macrophages was performed by ImageJ (Bethesda, MD). Immunofluorescence analyses were performed in naive, activated, and trained macrophages cultured in removable ibidi 18-well plates (Ibidi, Gräfelfing, Germany) suitable for microscopy that were fixed with a 4% paraformaldehyde solution 6h after LPS stimulation. Slides were blocked using PBS containing 1mM of CaCl_2_, 1mM of MgCl_2_, 20% of donkey serum (Jackson Immuno Researchlabs, West Grove, PA), and 0.3% of Triton X-100 (Merck Millipore) for 2h, and staining performed with rabbit anti-mouse CXCL10 (Thermo Fisher Scientific) and AF-647 rat anti-mouse SAA3 (BD Biosciences) antibodies. AF-488 donkey anti-rabbit IgG (Jackson ImmunoResearch) was used as a secondary antibody for CXCL10 detection. Slides were mounted using Prolong-Diamond (Thermo Fisher Scientific). Confocal Z-stack IF images were obtained on a Leica SP8 confocal microscope (Leica Microsystems) and composites were prepared by ImageJ (Bethesda, MD).

#### Flow cytometry

Purity of bone marrow monocytes isolated by negative selection was validated by flow cytometry before their use. Monocytes were stained with 1μg/mL of 4’,6-diamidino-2-phenylindole (DAPI, Thermo Fisher Scientific), blocked with anti-mouse CD16/32, and stained with monoclonal anti-mouse CD11b (M1/70), F4/80 (BM8), Ly6C (HK1.4), Ly6G (1A8), and MHC-II (M5/114.15.2) antibodies (all from BioLegend, San Diego, CA) at 4°C for 45 min. Purity analysis was determined in a LRS Fortessa cytometer (BD Biosciences). Phenotypic and functional characterization of naive, activated, and trained macrophages was performed by spectral flow cytometry. After culture, cells were washed with FACS buffer (2% FBS and 0.5mM EDTA in PBS), labeled for viability with LIVE/DEAD Fixable Blue, and Fc receptors blocked using anti-mouse CD16/32 (all from Thermo Fisher Scientific). Cells were stained with monoclonal anti-mouse CD11b (M1/70), F4/80 (BM8), Ly6C (HK1.4), Ly6G (1A8), MHC-II (M5/114.15.2), CD86 (GL-1), CD80 (16-10A1), CD40 (3/23), OX40L (RM134L), PD-L1 (10F.9G2), PD1 (29F.1A12), CD206 (C068C2), and ICOSL (HK5.3) antibodies (all from BioLegend, San Diego, CA) at 4 °C for 45min, fixed with 4% PFA, and acquired in a 5-laser Aurora cytometer (Cytek Biosciences). Cytobank platform (Cytobank, Santa Clara, CA) was used for dimensionality reduction of data obtained from macrophages analyses by performing a t-distributed stochastic neighbor embedding (t-SNE) analysis with perplexity and interaction values set at 30 and 1000, respectively.

#### T cell proliferation assay

Naive CD4 and CD8 T cells were isolated from the spleen of C57BL/6 mice by fluorescence-activated cell sorting (FACS) and labeled with 2.5 μM of CFSE CellTrace (Thermo Fisher Scientific). Naive, activated, and trained macrophages were cultured as described above and after 6h of LPS stimulation on day 6, cells were washed and macrophages were co-cultured with 2×10^5^ CFSE-labeled CD4 and CD8 T cells. After 5 days of culture cells were labeled for viability with 1μg/mL of DAPI (Thermo Fisher Scientific), stained with monoclonal anti-mouse CD3 (145-2C11), CD4 (RM4-5), and CD8 (53-6.7) antibodies (all from BioLegend) at 4°C for 45 min, and acquired in a LRS Fortessa (BD Biosciences). Analysis of CD4 and CD8 T cell proliferation was determined using FlowJo v10.10.0 software (BD Biosciences). Transwell experiments (Corning Inc.) were performed to determine cell contact-dependent T cell proliferation, placing macrophages on the basolateral compartment^89^ and CFSE-labeled CD4 and CD8 T cells on the apical side (top).

### QUANTIFICATION AND STATISTICAL ANALYSIS

#### RNA-Seq preprocessing

A total of 11 RNA-Seq libraries from naive (n = 4), activated (n = 3) and trained (n = 4) conditions were processed using the same pipeline for compatibility. Libraries were first evaluated for theirquality using FastQC (v0.11.8, RRID:SCR_014583).^100^ Trim Galore! (v0.6.6, RRID: SCR_011847) was used to remove adapter and repetitive sequences, as well as low quality reads (Phred score < 20).^101^ Reads were aligned to the mouse genome reference GRCm38.p6 using the STAR aligner (v2.7.5b).^102^ GENCODE release M25 was used as the transcriptome reference (RRID:SCR_014966). Alignment and obtention of gene-level read counts was performed with Salmon (v1.2.1, RRID: SCR_017036) for all libraries.^103^ Genes with less than 5 reads in total across all samples were filtered out of further analyses.

#### ATAC-Seq preprocessing

A total of 17 ATAC-Seq libraries from naive (n = 6), activated (n = 5) and trained (n = 6) conditions were processed using the same pipeline for compatibility. Libraries were sequenced on Illumina Hi-Seq 4000 (50bp paired-end reads). Reads were first evaluated for their quality using FastQC (v0.11.8, RRID:SCR_014583).^100^ Reads were trimmed for adapter sequences using Trim Galore! (v0.6.6, RRID:SCR_011847) and aligned to the mouse genome reference GRCm38.p6 using Bowtie2 (version 2.2.8, RRID: SCR_016368) with end-to-end mode, -sensitive and -X 2000 parameters.^104^ Reads aligned to mtDNA and non-canonical chromosome were removed. Picard (v2.2.4, RRID:SCR_006525) was used to remove duplicated reads. Post-filtering bam file for samples from the same experimental conditions were merged using the merge function from Samtools (v1.11), followed by peak calling using MACS (v2.1.0, RRID:SCR_013291) with parameters –nomodel –nolambda –keepdup all –slocal 10000.^105^ Peak summits were extended +/− 200bp, and quantification of reads in peaks was performed using BEDTools multicov (v2.29.2, RRID:SCR_006646). Peaks with less than 100 counts in total across all samples were filtered out as low signal regions. Coverage tracks were generated from bam files using deepTools (v3.2.1, RRID:SCR_016366) bamCoverage with parameters –normalizeUsingRPKM –binsize 10. Bigwig files were further normalized using the normalization factor calculated by DESeq2 using total counts in peak regions. Bigwigs from the same condition were merged together and uploaded to UCSC genome browser for visualization.

#### Differential expression and accessibility analysis

Differential expression analysis in RNA-Seq and differential accessibility in ATAC-Seq were performed using DESeq2 (v1.30.1, RRID: SCR_015687) R package.^106^ A gene is considered differentially expressed if the Benjamini-Hochberg adjusted p-value is less than 0.05 and the absolute log2 fold change is greater than 1. Volcano plots were adapted to show differential expression results, and the plots were generated using ggplot2 (v3.3.5, RRID:SCR_014601).^107^ A peak is considered differentially accessible if the Benjamini-Hochberg adjusted p-value is lessthan 0.05 and the absolute log2 fold change is greater than 1.5. Enrichment heatmaps and profile plots, generated by deepTools (v3.2.1, RRID:SCR_016366) with reference-point mode, were used to visualize differential accessible regions.^108^ Sequencce data have been deposited in the Gene Expression Omnibus (GEO) with accession number GSE290033 for RNA-seq, and GSE290032 for ATAC-seq.

#### Metabolomic analysis

Data acquisition and analysis was performed with the MassHunter Quantitative Analysis Software (Agilent Technologies). The mass spectrometry metabolomics data have been deposited in the National Metabolomics Data Repository (NMDR) with data track ID: 6139.

#### Statistical analysis

Statistical details and the exact number of n (number of biological or experimental replicates) for each specific experiment are described in each figure legend. All experiments were carried out at least two or three times, and the findings of all key experiments were reliably reproduced Data for all experiments represent the geometric mean and the standard error of the mean (SEM). When comparing two groups, differences between experimental groups were determined using an unpaired t-test. When comparing more than 2 groups, one-way ANOVA with Tukey’s multiple comparisons test was used for post-hoc analysis or Kruskal-Wallis test when data are not normally distributed. Statistical significance was determined by *p ≤ 0.05, **p ≤ 0.01, ***p ≤ 0.005, **** p ≤ 0.001. Statistical analyses were conducted using GraphPad Prism v9.1 software.

## Supplementary Material

1

SUPPLEMENTAL INFORMATION

Supplemental information can be found online at https://doi.org/10.1016/j.celrep.2025.116202.

## Figures and Tables

**KEY RESOURCES TABLE:** 

REAGENT or RESOURCE	SOURCE	IDENTIFIER

Antibodies		

Anti-mouse CD16/32 Monoclonal Antibody (93)	ThermoFisher Scientific	Cat# \14-0161-82; AB_467133
Anti-mouse CD11b Super Bright^™^ 436 (clone M1/70)	Thermo Fisher Scientific	Cat#62-0112-82; AB_2662385
Anti-mouse MHC-II (I-A/I-E) Brilliant Violet 605 (clone M5/114.15.2)	BioLegend	Cat#107639; AB_2565894
Anti-mouse CD86 Brilliant Violet 650 (clone GL-1)	BioLegend	Cat#105035; AB_11126147
Anti-mouse CD206 Brilliant Violet 785 (clone C068C2)	BioLegend	Cat#141729; AB_2565823
Anti-mouse ICOSL(CD275) PE (clone HK5.3)	BioLegend	Cat#107405; AB_2248797
Anti-mouse PD1L PE/Dazzle 594 (clone 10F.9G2)	BioLegend	Cat#124324; AB_2565639
Anti-mouse F4/80 PE-Cy5 (clone BM8)	BioLegend	Cat#123112; AB893482
Anti-mouse Ly6G PE-Cy7 (clone 1A8)	BioLegend	Cat#127618; AB_1866271
Anti-mouse OX40L Alexa Fluor 647 (clone RM134L)	BioLegend	Cat#108810; AB2207379
Anti-mouse Ly6C Alexa Fluor 700 (clone HK1.4)	BioLegend	Cat#128024; AB_10643270
Anti-mouse CD40 APC-Fire 750 (clone 3/23)	BioLegend	Cat#124632; AB2734194
Anti-mouse PD1 (CD279) APC-Fire 810 (clone 29F.1A12)	BioLegend	Cat#135252; AB_2910292
Anti-mouse CD80 BUV 661 (clone 16-10A1)	BD Biosciences	Cat#741515; AB_2870964
Anti-mouse CD3ε APC-Cy7 (clone 145-2C11)	BioLegend	Cat# 100330; AB_1877170
Anti-mouse CD4 APC (clone RM4-5)	Thermo Fisher Scientific	Cat#17-0042-82; AB_469323
Anti-mouse CD8 PE-Cy7 (clone 53-6.7)	Thermo Fisher Scientific	Cat#25-0081-82; AB_469584
Alexa Fluor^®^ 647 Rat Anti-Mouse SAA3 (JOR110A)	BD Biosciences	Cat#566652; AB _2869809
Anti-CXCL10 Recombinant Rabbit Monoclonal Antibody (10H11L3)	Thermo Fisher Scientific	Cat#701225; AB_2532429
Alexa Fluor^®^ 488 AffiniPure^™^ Donkey Anti-Rabbit IgG (H + L)	Jackson ImmunoResearch	Cat# 711-545-152; AB_2313584

Chemicals, peptides, and recombinant proteins		

Dulbecco's Modified Eagle's Medium (DMEM)	Corning	Cat#5515-013-CV
Dulbecco's Phospate Buffered Saline (10X)	Lonza	Cat#17-515Q
Ack Lysis Buffer	Gibco	Cat#A10492-01
Fetal Bovine Serum, HyClone ^™^	Gibco	Cat#10270106
HEPES (1M)	Gibco	Cat#11560496
MEM Non-essential Amino Acid Solution	Gibco	Cat#11350912
Sodium Pyruvate (100mM)	Gibco	Cat#12539059
Penicillin-Streptomycin	Lonza	Cat#09-757F
2-mercaptoethanol	Sigma-Aldrich	Cat#516732
M-CSF	Peprotech	Cat#315-02
Lipopolysaccharide from *Salmonella enterica* serotype abortus equi	Sigma-Aldrich	Cat#L5886
Lipopolysaccharide from *Escherichia coli* O111:B4	Sigma-Aldrich	Cat# #L2630
β-1,3-Glucan from *Euglena gracilis*	Sigma-Aldrich	Cat# tlrl-wgp
β-1,3-Glucan from *Saccharomyces cerevisiae*	InvivoGen	Cat#89862
β-1,3-Glucan from *Candida albicans*	Stothers et al.^38^	David L. Williams
CFSE CellTrace^™^	Thermo Fisher Scientific	Cat# C34554
Donkey serum	Jackson ImmunoResearch	Cat#017-000-001
KAPA pure beads	Roche	Cat#07983298001
Triton X-100 laboratory grade	Merk	Cat#X100-5ML
ProLong^™^ Diamond	Thermo Fisher Scientific	Cat# P36965
DAPI	ThermoFisher	Cat# D1306
EDTA	N\A	N\A
LIVE/DEAD^™^ Fixable Blue	Thermo Fisher Scientific	Cat#L23105

Critical commercial assays		

EasySep Mouse Monocyte Isolation Kit	Stem Cell Technologies	Cat# 19861
IL-6 Mouse Uncoated ELISA Kit	Thermo Fisher Scientific	Cat#88-7064-88
TNF alpha Mouse Uncoated ELISA Kit	Thermo Fisher Scientific	Cat#88-7324-88
Mouse CXCL10/IP-10/CRG-2 DuoSet ELISA	R&D Systems	Cat#DY466
ELLA SimplePlex mouse IL6,	Bio-thechne	SPCKB-MP-000466
ELLA SimplePlex mouse TNF	Bio-thechne	SPCKB-MP-000822
ELLA SimplePlex mouse CXCL10	Bio-thechne	SPCKB-MP-001485
Mouse SAA-3 ELISA	Merck	Cat#EZMSAA3-12K
Glycolysis Stress Test kit	Agilent	Cat#103020-100
Mito Stress Test kit	Agilent	Cat#103015-100
L-Lactate assay kit	Abcam	Cat#Ab65330
Qubit^™^ dsDNA Quantification Assay Kits	Thermo Fisher Scientific	Cat#Q32851
NEBNext^®^ Single Cell/Low Input RNA Library Prep Kit for Illumina.	New United Kingdom Biolabs	Cat#E6420L

Deposited data		

RNA sequencing data accession number	This paper	GSE290033
ATAC sequencing data accession number	This paper	GSE290032
Metabolomics DOI number	This paper	http://doi.org/10.21228/M8583B

Experimental models: Organisms/strains		

Mouse: C57BL/6J	The Jackson Laboratory	JAX: 000664
Mouse: B6.129S4-Mtortm1.2Koz/J	The Jackson Laboratory	JAX: 011009
Mouse: B6.129P2-Lyz2tm1(cre)Ifo/J	The Jackson Laboratory	JAX: 004781
Mouse: BALB/cJ	The Jackson Laboratory	JAX: 000651

Software and algorithms		

FlowJo Software	TreeStar	https://www.flowjo.com
GraphPad Prism v9.1	GraphPad software	https://www.graphpad.com
ImageJ		https://imagej.nih.gov/ij/
Seahorse Wave Desktop Software	Agilent	https://www.agilent.com/en/product/cell-analysis/real-time-cell-metabolic-analysis/xf-software

Other		

RNA-seq and ATAC-seq data preprocessing and analysis	This paper	https://bings.mssm.edu/

## Highlights

- Kinetics of cytokine production demonstrate the functional steps of trained immunity
- Serum amyloid A3 (SAA3) is a biomarker of trained macrophages
- Trained macrophages induce contact-dependent T cell proliferation *in vitro*
- Induction of training is conditioned by cell cryopreservation and mouse genetics
