# Supplementary material for: In vitro protocol demonstrating five functional steps of trained immunity in mice: Implications on biomarker discovery and translational research
Source: Cell Rep. Author manuscript; Available in PMC 2026 Jan 15. (PMC12805498; doi:10.1016/j.celrep.2025.116202)
Supplement: 1 [file NIHMS2120066-supplement-1.pdf]

Cell Reports, Volume *44*

Supplemental information

In vitro protocol demonstrating five functional steps of trained immunity in mice: Implications on biomarker discovery and translational research

Maria González-Pérez, Jana Baranda, Leticia Pérez-Rodríguez, Patricia Conde, Carlos de la Calle-Fabregat, Marcos J. Berges-Buxeda, Alexander Dimitrov, Javier Arranz-Herrero, Sergio Rius-Rocabert, Alessia Zotta, Ana Dopazo, Nikita Poddar, Xuedi Wang, Estanislao Nistal-Villán, Raphaël Duivenvoorden, Joren C. Madsen, David L. Williams, Dan Hasson, Daniel Lozano-Ojalvo, Florent Ginhoux, Luke A.J. O'Neill, and Jordi Ochando

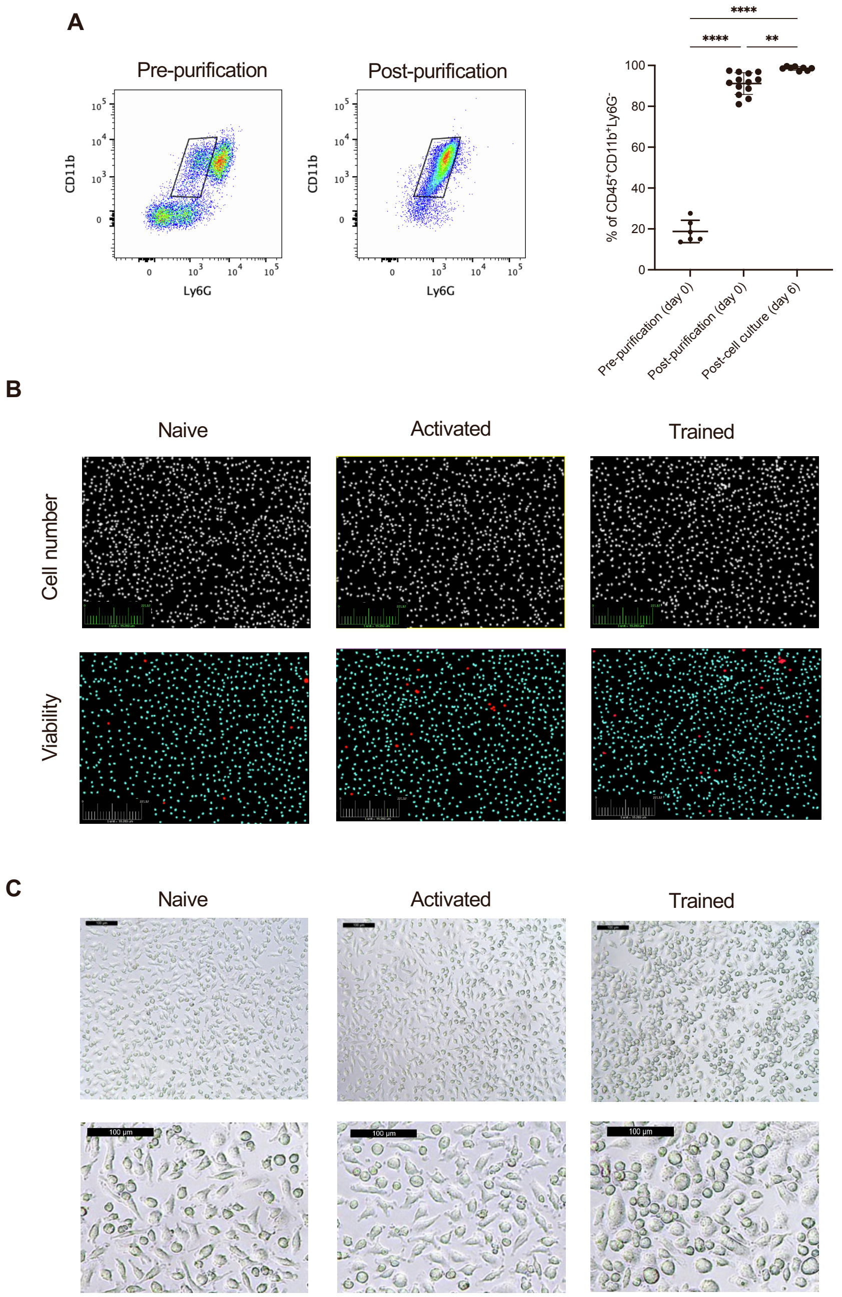

Figure S1. Isolation and morphological characterization of trained macrophages, Related to Figure 1

(A) Flow cytometry plots showing the frequency of $CD45^+CD11b^+Ly6G^-$ bone marrow monocytes before and after negative isolation with beads with a cell purity of 91.1 ± 5.3%. Data are presented as mean ± SEM (n = 8 independent experiments; paired t-test; $^{**}p \le 0.01$, $^{***}p \le 0.005$, $^{****}p \le 0.001$). **(B)** Representative images of nuclear staining with Hoechst (top) showing cell counts and cell viability of naive, activated and trained macrophages after LPS stimulation on day 6 (5x magnification). Live cells are shown in blue while dead cells are shown in red (bottom). **(C)** Representative phase contrast images of naive, activated, and trained macrophages showing cell morphology after LPS stimulation on day 6 (5x and 10x magnification).

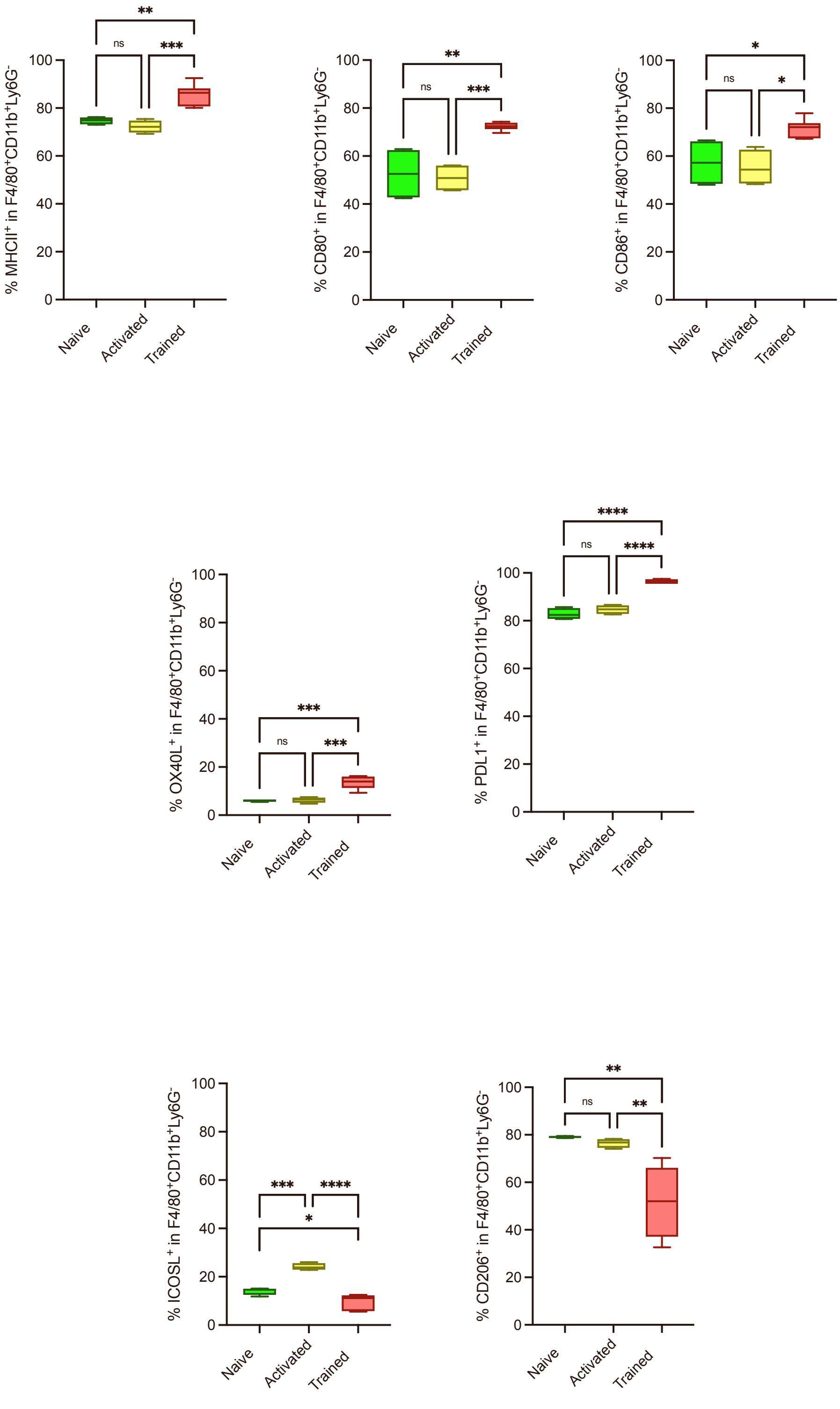

Figure S2. Phenotypic characterization of trained macrophages, Related to Figure 4

Percentage expression of MHC-II and costimulatory molecules CD80, CD86, OX40L, PDL1, ICOSL and CD206 in naive, activated and trained macrophages. Data are presented as mean ± SEM (n = 6 mice per group of two independent experiments; one-way ANOVA; *$p \le 0.05$, **$p \le 0.01$, ***$p \le 0.005$, ****$p \le 0.001$).
